# Phase I/II study of S-1 combined with paclitaxel in patients with unresectable and/or recurrent advanced gastric cancer

**DOI:** 10.1038/sj.bjc.6603497

**Published:** 2006-11-28

**Authors:** E Mochiki, T Ohno, Y Kamiyama, R Aihara, N Haga, H Ojima, J Nakamura, H Ohsawa, T Nakabayashi, K Takeuchi, T Asao, H Kuwano, and the North Kanto Gastric Cancer Study Group

**Affiliations:** 1 Department of General Surgical Science, Graduate School of Medicine, Gunma University, 3-39-22, Showa-machi, Maebashi, Gunma 371-8511, Japan; 2 Department of Surgery, Gunma Cancer Center Hospital, Gunma, Japan; 3 Department of Surgery, Saiseikai Maebashi Hospital, Gunma, Japan; 4 Department of Surgery, Ohmiya Red Cross Hospital, Saitama, Japan; 5 Department of Surgery, Fujioka General Hospital, Gunma, Japan; 6 Department of Surgery, Gunma Cardiovascular Center, Gunma, Japan; 7 Department of Surgery, Tone Chuo Hospital, Gunma, Japan

**Keywords:** advanced gastric cancer, S-1, paclitaxel

## Abstract

Both paclitaxel and S-1 are effective against gastric cancer, but the optimal regimen for combined chemotherapy with these drugs remains unclear. This phase I/II study was designed to determine the maximum tolerated dose (MTD), recommended dose (RD), dose-limiting toxicity (DLT), and objective response rate of paclitaxel in combination with S-1. S-1 was administered orally at a fixed dose of 80 mg m^−2^ day^−1^ from days 1 to 14 of a 28-day cycle. Paclitaxel was given intravenously on days 1, 8, and 15, starting with a dose of 40 mg m^−2^ day^−1^. The dose was increased in a stepwise manner to 70 mg m^−2^. Treatment was repeated every 4 weeks unless disease progression was confirmed. In the phase I portion, 17 patients were enrolled. The MTD of paclitaxel was estimated to be 70 mg m^−2^ because 40% of the patients given this dose level (two of five) had DLT. The RD was determined to be 60 mg m^−2^. In the phase II portion, 24 patients, including five with assessable disease who received the RD in the phase I portion, were evaluated. The median number of treatment courses was six (range: 1–17). The incidence of the worst-grade toxicity in patients given the RD was 28 and 8%, respectively. All toxic effects were manageable. The response rate was 54.1%, and the median survival time was 15.5 months. Our phase I/II trial showed that S-1 combined with paclitaxel is effective and well tolerated in patients with advanced gastric cancer.

Patients with unresectable and recurrent gastric cancer have extremely poor outcomes, with 5-year survival rates of less than 5% (Parker *et al*, 1997). Various chemotherapy regimens have been developed, but median survival in patients with unresectable or recurrent gastric cancer (or both) who receive chemotherapy remains less than 9–12 months (Wils *et al*, 1991; Kim *et al*, 1993; Koizumi *et al*, 2003; Ohtsu *et al*, 2003; Ajani *et al*, 2006b). Randomised phase III studies of combination chemotherapy for unresectable advanced gastric cancer reported median survival times (MSTs) of 5–9.6 months and overall response rates of 9–46% (Webb *et al*, 1997; Vanhoefer *et al*, 2000; Ross *et al*, 2002; Ohtsu *et al*, 2003). Combination chemotherapy thus appears to contribute only marginally to survival. New agents and combination chemotherapy regimens are needed to achieve greater survival benefits in far-advanced gastric cancer.

S-1, a fourth-generation oral fluoropyrimidine, is an oral formulation combining tegafur, 5-chloro-2,4-dihydroxypyridine, and potassium oxonate at a molar ratio of 1 : 0.4 : 1 (Takechi *et al*, 1997). S-1 is acknowledged to be a useful anticancer drug in Japan. Phase I and early phase II studies of S-1 as a single agent proposed 80 mg m^−2^ day^−1^ given orally for 28 consecutive days, followed by a 2-week rest period, as the tentative recommended dosage (Horikoshi *et al*, 1996; Sugimachi *et al*, 1999). A number of studies have reported that S-1 monotherapy is effective against gastric cancer, with response rates ranging from 26 to 53% (Horikoshi *et al*, 1996; Sakata *et al*, 1998; Koizumi *et al*, 2000; Chollet *et al*, 2003). The toxicity profile of S-1 in European studies apparently differs from that in Japanese studies, with more diarrhoea and hand-foot syndrome and less myelotoxicity (Chollet *et al*, 2003). In one early phase II clinical trial of S-1, the response rate was 53.6% (15 out of 28), and the median survival period was 298 days in Japanese patients with advanced gastric cancer (Horikoshi *et al*, 1996). Two late phase II studies of S-1 in advanced gastric cancer, which used similar treatment regimens, demonstrated high response rates of 49 and 44%, respectively (Sakata *et al*, 1998; Koizumi *et al*, 2000). These results suggested that S-1 is likely to become a key drug for the management of advanced gastric cancer.

Paclitaxel is a taxane derivative that was originally isolated from *Taxus brevifolia*, a type of Western yew (Wani *et al*, 1971). Paclitaxel, a newer taxane, has been shown to be effective against a variety of cancers, including breast cancer (Holmes *et al*, 1991), ovarian cancer (Einzig *et al*, 1992), and lung cancer (Chang *et al*, 1993). Paclitaxel is also an effective drug for gastric cancer, with response rates ranging from 20 to 28% in single-agent phase II studies (Ajani *et al*, 1998; Ohtsu *et al*, 1998; Yamada *et al*, 2001; Yamaguchi *et al*, 2002). Ajani *et al* (1998) reported that a 24-h infusion of paclitaxel is associated with a higher response rate and milder haematological toxicity than a 3-h infusion of an equivalent dose in patients with gastric cancer . The recommended optimal dose of paclitaxel in Japan was determined to be 210 mg m^−2^ once every 3 weeks (Yamaguchi *et al*, 2002). Recently, good results have been obtained with a weekly regimen of paclitaxel in patients with ovarian cancer and gastric cancer (Fennelly *et al*, 1997; Arai *et al*, 2003; Hironaka *et al*, 2006). The use of weekly regimens of paclitaxel, used mainly as second-line chemotherapy, has increased in Japan because of milder haematological toxicity as compared with regimens administering paclitaxel once every 3 weeks.

This multi-institution phase I/II study was designed to evaluate the efficacy and toxicity of combination therapy with paclitaxel, given in increasing doses, plus a fixed dose of S-1 (80 mg m^−2^) in patients with untreated, advanced gastric cancer. All participating centres belonged to the North Kanto Gastric Cancer Study Group.

## PATIENTS AND METHODS

### Patient eligibility

Eligible patients had histologically proved unresectable or recurrent gastric cancer. Up to one regimen of prior chemotherapy was allowed (adjuvant chemotherapy was allowed provided that at least 28 days had elapsed since the last treatment), except for prior treatment with taxanes (paclitaxel or docetaxel).

Other inclusion criteria were as follows: an age of 20–75 years; a performance status of 0–1 (Eastern Clinical Oncology Group); an estimated life expectancy of more than 3 months; a white blood cell count between 4000 and 12 000 mm^−3^; an absolute neutrophil count of over 2000 mm^−3^, a platelet count of over 100 000 mm^−3^, a haemoglobin level of over 8.0 g dl^−1^; aspartate aminotransferase and alanine aminotransferase levels within two times the upper limit of normal for the institution; a serum bilirubin level of less than 1.5 mg dl^−1^; a serum creatinine level within the upper limit of the normal value for the institution; a 24-h creatinine clearance of more than 50 ml min^−1^; and a normal electrocardiogram. Only patients who could swallow tablets were eligible. Patients were excluded if they had brain metastases, severe comorbid conditions, active double cancers, a past history of drug allergy, or were unable to comply with the protocol requirements. Pregnant women were also excluded. Written informed consent was obtained from all patients before study entry. This study was approved by the Ethics Committees at the participating sites.

### Treatment regimen and dose-escalation schedule

S-1 (Taiho Pharmaceutical Co., Ltd., Tokyo, Japan) was given orally twice daily after meals at a fixed dose of 80 mg m^−2^ day^−1^ for 14 consecutive days, followed by a 14-day rest period; this cycle was repeated every 4 weeks. The dose of S-1, decided on the basis of the patients' body surface area (BSA), was 40 mg (BSA <1.25 m^2^), 50 mg (BSA 1.25–1.5 m^2^), or 60 mg (BSA ⩾1.5 m^2^). Paclitaxel (Taxol; Bristol-Myers Squibb, Tokyo, Japan) was administered intravenously over the course of 60 min on days 1, 8, and 15. To prevent hypersensitivity reactions, all patients received 20 mg of dexamethasone intravenously, 50 mg of diphenhydramine orally, and 50 mg of ranitidine intravenously 1 h before paclitaxel infusion. The initially administered dose of paclitaxel was 40 mg m^−2^ (dose level 1). The dose was scheduled to be increased in 10 mg m^−2^ increments to 70 mg m^−2^ (dose level 4), unless the maximum tolerated dose (MTD) was reached. At least three patients received each dose level. If one of the three patients at a given dose had any dose-limiting toxicity (DLT), three other patients were assigned to receive the same dose. If one of the resulting six patients had DLT, the dose could be increased to the next level; if two or more patients had DLT, that level was deemed the MTD. If DLT occurred in two or all three of the patients initially assigned to a given dose level, that level was also considered the MTD. The MTD was thus defined as the dose at which >33% of the patients had DLTs during the first course of treatment. The recommended dose (RD) for phase II studies was defined as one level below the MTD. Toxicity was graded according to the National Cancer Institute common toxicity criteria, version 2.0. Dose-limiting toxicity was defined as follows: grade 4 leucopenia/neutropenia, febrile grade 3 neutropenia lasting for more than 4 days, grade 3 thrombocytopenia, and any grade 3 or above non-haematological toxicity (excluding anorexia, nausea, vomiting, alopecia, and general fatigue).

### Response evaluation and toxicity

Tumour response was evaluated by computed tomographic (CT) scans for each course of treatment. Imaging studies were repeated to confirm response at least 4 weeks (for complete or partial responses) after first documenting a given response. Thereafter, tumour response was evaluated by CT scanning every two cycles. Radiographs of all assessable patients were also reviewed externally to confirm investigator-designated responses. Tumour response was objectively evaluated according to guidelines for the evaluation of the response of solid tumours to treatment (Therasse *et al*, 2000).

### End points and statistical analysis

The primary study end point was the definition of the MTD and DLT of the described regimen of TS-1 plus paclitaxel. The MTD and recommended dose were further examined in additional patients to confirm their toxicity/safety profile and to ensure their suitability for future phase II trials. Additional study end points were to determine (i) the objective response rate according to the RECIST criteria; (ii) progression-free survival (calculated from the date of starting treatment to the date of progression or relapse); and (iii) overall survival. Survival from the date of study entry was estimated using the Kaplan–Meier product-limit method.

The calculation of sample size for the phase II portion of the study was based on a target activity level of 60% and a minimum activity level of 30%, at a significance level of 0.05 and *β* error=0.1. The required number of patients was estimated to be 21.

## RESULTS

### Patients' characteristics

Between August 2002 and April 2005, a total of 36 patients were enrolled. In the phase I part, 17 patients were studied between August 2002 and January 2004. The patients' characteristics are summarised in Table 1. Their median age was 62 years (range: 44–71 years). One patient had undergone a prior gastrectomy, and another patient had received adjuvant chemotherapy with S-1 alone before study entry. Histologically, 12 patients had intestinal-type adenocarcinoma, and five patients had diffuse-type adenocarcinoma. The sites of metastasis were the liver in two patients, the lymph nodes in 15, and the peritoneum in five. One patient (level 3) had a non-measurable metastatic lesion in the peritoneum.

In the phase II part of the study, 19 patients were enrolled between August 2004 and April 2005 (Table 1). Their median age was 63 years (range: 48–75 years). Four patients had undergone a prior gastrectomy and received adjuvant chemotherapy with S-1 alone before study entry. Histologically, 13 patients had intestinal-type adenocarcinoma, and six patients had diffuse-type adenocarcinoma. All 19 patients had measurable metastatic lesions, involving the lymph nodes in 14 patients and the liver in six. Nine patients (47%) had non-measurable metastatic lesions in the peritoneum.

A total of 201 courses of treatment were given. The median number of treatment courses was four (range: 1–13) and six (range: 1–17) in the phase I and II portions, respectively. The median duration of therapy per patient was 251 days (range: 28–523) in the phase II portion.

### Determination of MTD

Toxic effects are summarised in Table 2. In the phase I part of the study, all patients were evaluated for adverse reactions, and 16 completed one or more cycles of treatment. None of the six patients given dose level 1 or 2 had DLT. At level 3, one patient had grade 3 leucopenia during the first cycle, and another had grade 3 anaemia during the second. Three other patients were then assigned to receive dose level 3 to reconfirm safety; none had DLT. At level 4, one patient had grade 3 anorexia (20%) with grade 3 diarrhoea (20%) and vertigo (20%) during the first cycle, whereas another had grade 3 hyperkalemia (20%). The plasma potassium concentration increased after treatment with paclitaxel and then gradually fell to the normal range. Vertigo occurred 30–40 min after the administration of paclitaxel. These toxic effects repeatedly occurred after treatment with paclitaxel and resolved completely without any medication. They were therefore considered treatment-related. Dose-limiting toxicity occurred in two of the five patients given dose level 4. On the basis of these results, dose level 4 was considered the MTD, and dose level 3 was determined to be the RD.

### Efficacy

In the phase I portion, one of the 17 patients had no measurable lesions. Efficacy was evaluated in the other 16 patients. Seven patients had a partial response (PR), two had stable disease (SD), and seven had progressive disease (PD), yielding a response rate of 43.7% in patients with assessable lesions (Table 3). The response rate was 36.3% (four out of 11) in the patients with intestinal-type adenocarcinoma and 60% (three out of five) in those with diffuse-type adenocarcinoma (Table 3).

**Figure 1:**
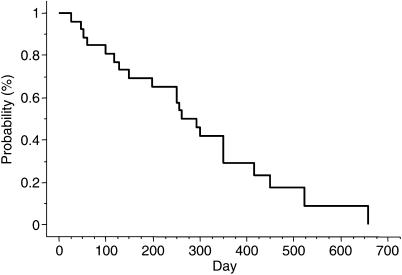
Cumulative probability of progression-free survival as estimated by the Kaplan–Meier method in 24 patients.

**Figure 2:**
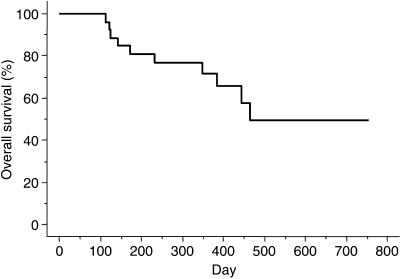
Cumulative probability of overall survival as estimated by the Kaplan–Meier method in 24 patients.

Twenty-four patients, including five enrolled in the phase I portion of the study, were evaluated to determine the response rate at the RD in the phase II portion. The overall response rate was 54.1% (95% confidence interval (CI): 34.2–74.1%); one patient had a complete response (CR), 12 had PR, six had SD, and five had PD (Table 3). The response rate according to pathological type was 58.8% (10 out of 17) for intestinal-type adenocarcinoma and 42.8% (three out of seven) for diffuse type. The median overall duration of response in the 13 responders in the phase II portion was 7.8 months (95% CI, 4.2–10.1 months). The median time to progression (TTP) was 9.5 months (95% CI, 5–11.6 months) in the phase II portion (Figure 1). Median survival time in the phase II portion was 15.5 months (95% CI, 11.6–19.4 months), and the 1- and 2-year survival rates were 71.7 and 49.3%, respectively (Figure 2).

### Toxicity

In the phase II portion, the median number of courses administered was six (range: 1–17). In the 25 patients who received the RD, including the six patients assigned to level 3 in the phase I portion, the most frequent types of severe (grades 3 and 4) haematological toxicity were leucopenia (five cases, 20%), neutropenia (five cases, 20%), and thrombocytopenia (four cases, 16%), as shown in Table 4. The most common types of non-haematological toxicity were stomatitis (seven cases, 28%), nausea (five cases, 20%), and rash (five cases, 20%). Among the 25 patients in the phase II portion, the dose of paclitaxel was reduced in three patients within one cycle because of neutropenia and thrombocytopenia. The incidence of the worst-grade toxicity in patients given the RD was none in seven patients (28%), grade 1 in four (16%), grade 2 in five (20%), grade 3 in seven (28%), and grade 4 in two (8%). There was no treatment-related death or delayed severe toxicity.

## DISCUSSION

This study was undertaken to determine the RD for phase II studies of paclitaxel combined with S-1 for advanced metastatic gastric cancer and to investigate the antitumour effect and feasibility of this combination. The RD was determined to be 60 mg m^−2^ of paclitaxel on days 1, 8, and 15 plus 80 mg m^−2^ day^−1^ of S-1 on days 1–14 of a 28-day cycle. The response rate at the RD was 54.1%, with an MST of 15.5 months, and a TTP of 9.5 months. These are promising results in patients with advanced metastatic gastric cancer. Moreover, toxicity was not severe, and therapy could be administered on an outpatient basis.

Two late phase II trials of S-1 therapy in Japanese patients with advanced gastric cancer have reported a high overall response rate of 44–49% and an MST of 7–8 months (Sakata *et al*, 1998; Koizumi *et al*, 2000). These findings suggest that S-1 is one of the most effective currently available antitumour agents for gastric cancer. To further enhance efficacy and improve survival, several ongoing studies are assessing the response to S-1 combined with other anticancer agents with different mechanisms of action. Clinically, paclitaxel has been used in combination with 5-fluorouracil (5-FU) and its derivatives for several reasons. First, paclitaxel is considered an effective drug for gastric cancer, with response rates ranging from 20 to 28% in single-agent phase II studies (Ajani *et al*, 1998; Ohtsu *et al*, 1998; Yamada *et al*, 2001; Yamaguchi *et al*, 2002). Furthermore, Ajani *et al* (2006a) reported that sequential treatment with paclitaxel and bryostatin-1 is effective, with a response rate of 29%. Paclitaxel is a good candidate for combined treatment because of it lacks cross-resistance to fluoropyrimidine derivatives. S-1 and paclitaxel have different mechanisms of action, and the principal toxicities of these drugs do not overlap (Fujitani *et al*, 2005). In recent years, weekly regimens of paclitaxel, based on its mechanism of action, have been widely studied. One study comparing a weekly infusion of paclitaxel with an infusion once every 3 weeks documented similar efficacy with decreased adverse reactions (in particular, myelosuppression and peripheral neuropathy) with the weekly regimen in women with ovarian cancer (Andersson *et al*, 2000). A combination of paclitaxel and 5-FU has been demonstrated to have additive cytotoxicity against tumour cell lines *in vitro*, especially strong with sequential exposure (Kano *et al*, 1996). Moreover, Kondo *et al* (2005) reported a MST of 335 days in a phase I study of weekly paclitaxel plus 5-FU in patients with advanced gastric cancer. The second reason for combining paclitaxel with 5-FU is that their principal toxicities differ considerably. Neuropathy and neutropenia are the principal toxicities of paclitaxel, whereas stomatitis and diarrhoea are the predominant toxicities of fluoropyrimidines in most commonly used regimens (Bokemeyer *et al*, 1997; Cascinu *et al*, 1998). We therefore combined paclitaxel with S-1.

As most toxic effects of S-1 occurred after 4 consecutive weeks of treatment in phase II studies, S-1 was administered daily at a fixed dose of 80 mg m^−2^ for 2 consecutive weeks in combination with paclitaxel on days 1, 8, and 15 of a 28-day cycle. This duration of treatment with S-1 was 2 weeks shorter than the time at which toxicity such as leucopenia generally appears. We studied this regimen in a phase I/II trial in patients with unresectable or recurrent advanced gastric cancer.

The main objective of our phase I study was to evaluate the safety and optimal dose of paclitaxel in combination with S-1. The incidences of grades 3 and 4 toxic effects in our study are consistent with those reported previously for the same S-1 combination regimen (Ajani *et al*, 2006b; Inokuchi *et al*, 2006). When paclitaxel was administered every 3 weeks with or without 5-FU, non-haematological toxicity, apart from alopecia of grade 3 or higher, included neuropathy (0–29%), myalgia (0–27%), and nausea/vomiting (0–7%) (Ohtsu *et al*, 1998; Yamada *et al*, 2001; Arai *et al*, 2003; Hokita *et al*, 2005; Kondo *et al*, 2005; Inokuchi *et al*, 2006). Although there are limitations in comparing the results of different studies because of differences in factors such as the dose and treatment schedule of paclitaxel therapy and the extent of prior treatment, neuromuscular toxicity, the most common adverse effect of paclitaxel, was very mild in our study. The good tolerability of our regimen is reflected in the fact that patients received a median of six treatment courses in the phase II portion.

An MST of at least 12 months and a TTP of at least 8 months would strongly suggest a significant advance in the treatment of advanced gastric cancer (Ajani *et al*, 2006b). In our phase I/II study, the response rate was 54.1% and the MST was 15.5 months. The MST in our study was longer than that in previous phase II studies of S-1 as a single agent (207–250 days) (Sakata *et al*, 1998; Koizumi *et al*, 2000). Our response rate is equivalent to that reported for TS-1/cisplatin therapy (Koizumi *et al*, 2003). Moreover, the MST in our study was longer than the 383 days obtained in a previous study of S-1 combined with cisplatin (Koizumi *et al*, 2003). The incidences of grade 3 or 4 haematological and non-haematological toxicity were similar to those with S-1 plus cisplatin (Koizumi *et al*, 2003; Ajani *et al*, 2006b). The high response rate and longer MST in our study may be related to the better performance status of our patients as compared with that in other studies. With cisplatin-based regimens, patients must receive intravenous infusions to ensure adequate hydration and prevent cisplatin-induced renal damage. S-1 combined with paclitaxel might therefore be better suited for treatment on an outpatient basis than cisplatin-based chemotherapy. A high response rate coupled with a better quality of life is considered an advantage of S-1 plus paclitaxel.

In 2005, Hokita *et al* (2005) reported the results of a phase I study of S-1 combined with paclitaxel in patients with advanced gastric cancer. The response rate was 53%, and the MST was 428 days. Furthermore, Fujitani *et al* (2005) reported a preliminary response rate of 62.5% (five out of eight) in their phase I study of S-1 plus weekly paclitaxel in patients with advanced gastric cancer. These response rates and MST are comparable to those in our study. A variety of taxane-based combination chemotherapy regimens have been developed for advanced gastric cancer, steadily improving response rates. Ajani *et al* (2005) reported that a combination of docetaxel, cisplatin, and fluorouracil is highly active against advanced untreated gastric or gastroesophageal adenocarcinoma. Docetaxel has also been combined with TS-1, and available evidence indicates that this combination is effective and well tolerated in patients with advanced gastric cancer (Yamaguchi *et al*, 2006).

In conclusion, our phase I/II trial suggests that S-1 combined with paclitaxel is effective and well tolerated. Because this was a small, uncontrolled study, phase III trials of S-1 plus paclitaxel are needed to confirm benefits in terms of survival and quality of life in patients with advanced gastric cancer, thereby establishing the value of this new regimen. At present, a randomised study comparing TS-1 plus paclitaxel with TS-1 plus cisplatin is being carried out by the North Kanto Gastric Cancer Study Group.

## Conflict of interest statements

E Mochiki, T Ohno, Y Kamiyama, R Aihara, N Haga, J Ojima, J Nakamura, H Ohsawa, T Nakabayashi, T Asao, and H Kuwano hereby declare not to be engaged in any financial or personal relationship with other people or organisations that could inappropriately influence their work.

## Figures and Tables

**Table 1:** Patients' characteristics

	**Phase I (*n*=17)**	**Phase II (*n*=19)**
Median age, years (range)	64 (44–71)	63 (48–75)
Male/female	12/5	15/4
		
*Performance status*		
0	14	15
1	3	4
		
*Histological type*		
Intestinal	12	13
Diffuse	5	6
		
*Prior therapy*		
None	15	15
Gastrectomy	1	0
Gastrectomy+chemotherapy	1	4
		
*Site of metastasis*		
Liver	2	6
Lymph nodes	15	14
Peritoneum	5	0

**Table 2:** Toxic effects and number of patients with toxicity according to the dose level of paclitaxel

**Paclitaxel**	**Level 1 (*n*=3)**		**Level 2 (*n*=3)**		**Level 3 (*n*=6)**		**Level 4 (*n*=5)**	
**Grade**	**1–2**	**3–4**	**1–2**	**3–4**	**1–2**	**3–4**	**1–2**	**3–4**
*Haematological*								
Leucopenia	2	0	1	0	3	1	0	0
Neutropenia	2	0	0	0	2	0	1	0
Anaemia	1	0	2	0	2	1	1	0
Thrombocytopenia	0	0	0	0	0	0	0	0
								
*Non-haematological*								
Anorexia	0	0	1	0	2	0	0	1
Nausea	1	0	0	0	1	0	1	0
Diarrhoea	0	0	0	0	1	0	0	1
Fatigue	0	0	0	0	2	0	1	0
Stomatitis	1	0	0	0	0	0	0	0
Rash	0	0	0	0	0	0	1	0
Vertigo	0	0	0	0	0	0	0	1
Bilirubin	0	0	0	0	0	0	1	0
AST/ALT	0	0	0	0	0	0	2	0
Hyperkalemia	0	0	0	0	0	0	0	1

AST=aspartate aminotransferase; ALT=alanine aminotransferase; *n*=number of patients.

National Cancer Institute common toxicity criteria (version 2).

**Table 3:** Overall response to treatment

	***n***	**CR**	**PR**	**SD**	**PD**	**NE**	**RR (%)**
*Phase I*							
Overall	17	0	7	2	7	1	43.7
*Level*							
1	3	0	1	0	2	0	33.3
2	3	0	2	1	0	0	66.6
3	6	0	2	1	2	1	50.0
4	5	0	2	0	3	0	40.0
*Histological type*							
Intestinal	12	0	4	2	5	1	36.3
Diffuse	5	0	3	0	2	0	60.0
							
*Phase II* a							
Overall	24	1	12	6	5	0	54.1
*Histological type*							
Intestinal	17	1	9	3	4	0	58.8
Diffuse	7	0	3	3	1	0	42.8

CR=complete response; PR=partial response; SD=stable disease; PD=progressive disease; NE=not evaluated; RR=response rate.

a Including five patients assigned to level 3 in the phase I portion.

**Table 4:** Adverse events observed in 25 patients

	**Number of patients (%)**		
**Adverse events**	**Grade 3**	**Grade 4**	**Grade 3 or 4**
*Haematological*			
Leucopenia	5	0	5 (20)
Neutropenia	3	2	5 (20)
Anaemia	2	0	2 (8)
Thrombocytopenia	4	0	4 (16)
			
*Non-haematological*			
Anorexia	2	0	2 (8)
Nausea	4	1	5 (20)
Diarrhoea	1	0	1 (4)
Fatigue	2	0	2 (8)
Stomatitis	6	1	7 (28)
Rash	5	0	5 (20)

National Cancer Institute common toxicity criteria (version 2).
